# Thermal Stability Kinetics and Shelf Life Estimation of the Redox-Active Therapeutic and Mimic of Superoxide Dismutase Enzyme, Mn(III) *meso*-Tetrakis(*N*-ethylpyridinium-2-yl)porphyrin Chloride (MnTE-2-PyPCl_5_, BMX-010)

**DOI:** 10.1155/2021/7003861

**Published:** 2021-12-06

**Authors:** Clarissa G. C. Maia, Bárbara C. R. de Araujo, Maria B. de Freitas-Marques, Israel F. da Costa, Maria Irene Yoshida, Wagner da Nova Mussel, Rita de Cássia O. Sebastião, Júlio S. Rebouças

**Affiliations:** ^1^Departamento de Química, Centro de Ciências Exatas e da Natureza, Universidade Federal da Paraíba, João Pessoa, PB 58051-900, Brazil; ^2^Departamento de Química, Instituto de Ciências Exatas, Universidade Federal de Minas Gerais, Belo Horizonte, MG 31207-901, Brazil

## Abstract

Mn(III) *meso*-tetrakis(*N*-ethylpyridinium-2-yl)porphyrin chloride (MnTE-2-PyPCl_5_, BMX-010, and AEOL10113) is among the most studied superoxide dismutase (SOD) mimics and redox-active therapeutics, being currently tested as a drug candidate in a phase II clinical trial on atopic dermatitis and itch. The thermal stability of active pharmaceutical ingredients (API) is useful for estimating the expiration date and shelf life of pharmaceutical products under various storage and handling conditions. The thermal decomposition and kinetic parameters of MnTE-2-PyPCl_5_ were determined by thermogravimetry (TG) under nonisothermal and isothermal conditions. The first thermal degradation pathway affecting Mn-porphyrin structural integrity and, thus, activity and bioavailability was associated with loss of ethyl chloride via *N*-dealkylation reaction. The thermal stability kinetics of the *N*-dealkylation process leading to MnTE-2-PyPCl_5_ decomposition was investigated by using isoconversional models and artificial neural network. The new multilayer perceptron (MLP) artificial neural network approach allowed the simultaneous study of ten solid-state kinetic models and showed that MnTE-2-PyPCl_5_ degradation is better explained by a combination of various mechanisms, with major contributions from the contraction models R1 and R2. The calculated activation energy values from isothermal and nonisothermal data were about 90 kJ mol^–1^ on average and agreed with one another. According to the R1 modelling of the isothermal decomposition data, the estimated shelf life value for 10% decomposition (*t*_90%_) of MnTE-2-PyPCl_5_ at 25°C was approximately 17 years, which is consistent with the high solid-state stability of the compound. These results represent the first study on the solid-state decomposition kinetics of Mn(III) 2-*N*-alkylpyridylporphyrins, contributing to the development of this class of redox-active therapeutics and SOD mimics and providing supporting data to protocols on purification, handling, storage, formulation, expiration date, and general use of these compounds.

## 1. Introduction

Water-soluble, cationic Mn(III) porphyrins derived from the 2-*N*-pyridylporphyrin scaffold were originally developed as potent superoxide dismutase (SOD) mimics, peroxynitrite scavengers, and later proven to be efficient redox-active therapeutics [1–6]. Two lead compounds, Mn(III) *meso*-tetrakis(*N*-ethylpyridinium-2-yl)porphyrin chloride (MnTE-2-PyPCl_5_, also known as BMX-010) [2] and Mn(III) *meso*-tetrakis(*N*-n-butoxyethylpyridinium-2-yl)porphyrin chloride (MnTnBuOE-2-PyPCl_5_, also known as BMX-001) [7, 8], were found to be safe and well tolerated in phase I clinical trials [1, 9–13]. Whereas MnTE-2-PyPCl_5_ progressed to various phase II clinical trials on atopic dermatitis and itch, the MnTnBuOE-2-PyPCl_5_ analogue, which was developed based on the MnTE-2-PyPCl_5_ prototype, is now being investigated in four phase II clinical trials as radioprotectors for glioma, head and neck cancer, multiple brain metastases, and anal squamous cell carcinoma [1, 10, 11, 14].

MnTE-2-PyPCl_5_ has an established reputation of being the Mn(III) 2-*N*-alkylpyridylporphyin-based prototype for the design of bioavailable SOD mimics, the development of catalytic antioxidants, and mechanistic studies [1, 3, 12, 15–17]. Given its safe toxicity profile in animal models and humans [1, 9, 10, 13], MnTE-2-PyPCl_5_ is usually the compound of choice in most exploratory preclinical studies [12, 17–25], being forwarded to translational medicine and clinical trials [1, 9, 10], and is now recognized as an excellent redox-active therapeutic [1], with well-defined pharmacokinetics [26, 27]. Despite the large number of studies dedicated to unravelling the biological and clinical aspects of MnTE-2-PyPCl_5_ both in vitro and in vivo [1, 16–18, 22], studies on the stability of this class of compounds are still somewhat limited [28–30]. It is worth noting, however, that information on MnTE-2-PyPCl_5_ stability may shed light on the stability of other MnP analogues [28] and likely support protocols on purification, handling, storage, formulation, expiration date, and general use of these compounds [31–34].

**Figure 1:**
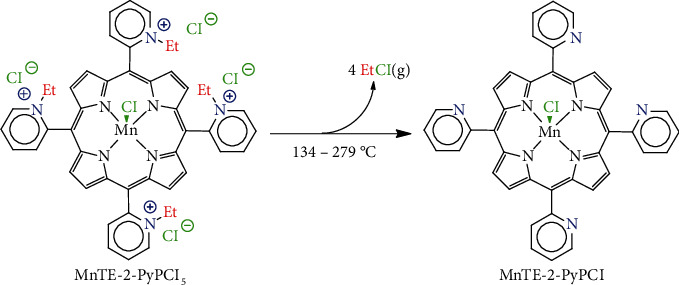
Thermal *N*-dealkylation of MnTE-2-PyPCl_5_ under dynamic air during thermogravimetric analysis at a heating rate of 10°C/min.

As a characteristic of Mn-porphyrins in general, MnTE-2-PyPCl_5_ with Mn in the +3 oxidation state is extremely stable against acid solvolysis: Mn(III) demetallation is difficult even under concentrate H_2_SO_4_ conditions [2]. MnTE-2-PyPCl_5_ is relatively stable against oxidative degradation under biological conditions, particularly the presence of many reactive substrates or biological sacrificial reductors [35]. Not only do Mn-porphyrins withstand major reactive oxygen and nitrogen species (ROS/RNS) but also H_2_O_2_ is a key molecule in the therapeutic effects of Mn-porphyrins, as clearly shown by Jaramillo et al. [36] and recently summarized elsewhere [1]. Whereas MnTE-2-PyPCl_5_ undergoes some bimolecular degradation when incubated with net H_2_O_2_ [22, 29], decomposition by peroxides is not at all an issue for solid-state/aqueous solution storage and/or general formulation of this compound [28]. The thermal degradation of MnTE-2-PyPCl_5_, as a representative of the Mn(III) 2-*N*-alkylpyridylporphyrin class, has been addressed in a single thermogravimetric study so far [28]. The thermal behavior of MnTE-2-PyPCl_5_ salts under aerobic conditions is characterized by three events: (i) dehydration of associated water molecules (room temperature to 134°C), (ii) *N*-dealkylation via loss of ethyl chloride (134–279°C) to yield MnT-2-PyPCl (see Figure 1), and (iii) various overlapping porphyrin-ring decomposition processes to yield manganese oxides as combustion residues (279–950°C) [28]. As MnTE-2-PyPCl_5_ is often administered as aqueous formulations by various routes (e.g., intraperitoneal, subcutaneous, intravenous, and oral) [17, 26, 27], MnP intrinsic biological activity on the Mn basis is not affected by reversible dehydration [28]. Conversely, *N*-dealkylation of MnTE-2-PyPCl_5_ changes the porphyrin structure irreversibly, reducing the overall charge of the molecule, which affects Mn(III)/Mn(II) redox potential, electrostatic facilitation for superoxide/peroxynitrite approach/scavenging, reactions with protein thiolates, and MnTE-2-PyP^5+^(aq) pharmacological activity [1, 17, 37, 38].

Thermogravimetric (TG) analyses are important for the evaluation and/or comparison of thermal stabilities of pharmaceutical materials, the study of drug-excipient compatibility in drug products, and the determination of kinetic parameters associated with thermal processes (such as activation energies, frequency factor, and reaction order) [31–34, 39, 40]. Overall, this information is useful for the development of pharmaceutical products and quality control [32, 34, 39, 41]. Studies on the kinetics of thermal degradation are recommended to evaluate the thermal stability of active pharmaceutical ingredients (API) and/or their solid-state pharmaceutic formulations [31–34, 39, 41, 42]. These kinetic studies are often carried out in the pharmaceutic field by nonisothermal (dynamic) or isothermal thermogravimetric analyses [43–49]. Kinetic studies under isothermal TG conditions are particularly relevant as they are amenable to Arrhenius equation treatment and make possible the estimation of room temperature degradation rate from extrapolation of accelerated, experimental, high-temperature degradation rates [34, 42]. These kinetic studies are particularly useful to provide reliable estimates of the shelf life of pharmaceutical products under various storage and handling conditions [31, 34].

We describe herein the first study on the kinetics of thermal degradation of MnP-based redox-active therapeutics under isothermal and nonisothermal conditions. Additionally, it is worth noting that thermal decomposition kinetic models were evaluated using a state-of-the-art approach, based on the multilayer perceptron (MLP) artificial neural network [48–52]. MnTE-2-PyPCl_5_ was chosen as a representative API of the class of Mn(III) 2-*N*-alkylpyridylporphyrins, currently developed as SOD mimics, biomimetic models, catalytic antioxidants, and redox-active therapeutics.

## 2. Materials and Methods

MnTE-2-PyPCl_5_ and the nonalkylated analogue MnT-2-PyPCl (see Figure 1) were prepared and purified as previously reported [2, 53] and showed characterization features consistent with published data [28, 53–55]. Although these compounds are usually labeled as anhydrous species (i.e., MnTE-2-PyPCl_5_), the isolated solids often contain variable amounts of water molecules, depending on sample workup, handling, and storage [28]. In this work, the cationic MnP, MnTE-2-PyPCl_5_, contained 8 molecules of water, whereas the neutral MnP, MnT-2-PyPCl, was analyzed as an anhydrous species, according to thermogravimetric data (see below). All samples were investigated by simultaneous thermogravimetric and differential thermal analyses (TG/DTA) under isothermal and nonisothermal conditions. The experimental kinetic designs described below for these two conditions of analyses agree with the International Confederation for Thermal Analysis and Calorimetry (ICTAC) recommendations [45–47].

### 2.1. Thermal Analysis

TG curves were obtained on a Shimadzu DTG60 thermal analyzer using 2–3 mg samples, accurately weighed in alumina crucibles directly in the thermobalance, under a dynamic atmosphere of synthetic air with a flow rate of 50 mL min^–1^.

For the kinetic studies, various TG curves were registered independently using heating rates of 5, 7.5, 10, and 12.5°C min^–1^ in the temperature range from 30 to 600°C for nonisothermal experiments. Isothermal experiments were carried out at 158, 160, 162, and 164°C for a period of 60 minutes; in these experiments, the samples were heated from room temperature to the target isothermal temperature with a heating rate of 10°C min^–1^.

### 2.2. Kinetic Study Using the Vyazovkin Isoconversional Method in Nonisothermal TG Data

The solid-state decomposition process follows the general first-order kinetic equation:

(1) $$\begin{array}{c} \frac{d\alpha }{dT}=k\left(T\right)f\left(\alpha \right), \end{array}$$

with *k*(*T*) as the rate constant following the Arrhenius equation and *f*(*α*) as the reaction model, with *α* as the conversion degree; *α* = (*m*_0_ − *m*_t_)/(*m*_0_ − *m*_f_), where *m*_0_ is the initial sample mass, *m*_f_ is the final sample mass, and *m*_*t*_ is the sample mass at time *t*.

Assuming *β*_*i*_(*i* = 1, ⋯, *n*) as the heating rate, the temperature integral *I*(*E*, *T*) = ∫_0_^*T*^exp(−*E*_*α*_/RT)*dt*, and (*α*) = ∫_0_^*α*^[*f*(*α*)]^−1^*dα*, the general equation (1) can be rewritten as

(2) $$\begin{array}{c} g\left(\alpha \right)=\frac{A}{\beta }\;I\left(E,T\right). \end{array}$$

The isoconversional Vyazovkin [44] method assumes that the reaction model is independent of the heating rate; for this, we have for a given conversion

(3) $$\begin{array}{c} \left(\frac{A_{\alpha }}{\beta_{1}}\right)I\left(E_{\alpha },T_{\alpha 1}\right)=\left(\frac{A_{\alpha }}{\beta_{2}}\right)I\left(E_{\alpha },T_{\alpha 2}\right)=\cdots =\left(\frac{A_{\alpha }}{\beta_{n}}\right)I\left(E_{\alpha },T_{\alpha n}\right). \end{array}$$

The frequency factor, *A*_*α*_, is considered constant for the processes submitted to small variation of the heating rate, so this equation can be treated considering the following optimization:

(4) $$\begin{array}{c} \overset{n}{\underset{i=1}{\sum }}\overset{n}{\underset{j\neq 1}{\sum }}\frac{\text{I}\left(E_{\alpha },T_{\alpha 1}\right)\beta_{j}}{\text{I}\left(E_{\alpha },T_{\alpha 1}\right)\beta_{i}}=\text{minimum}. \end{array}$$

The temperature integral can be solved assuming the following approximation [56]:

(5) $$\begin{array}{c} \int_{0}^{T}\exp\left(\frac{-E}{\text{RT}}\right)dT=\frac{E}{R}\int_{\infty }^{x}\frac{\exp\left(-x\right)}{x^{2}}dx=\frac{E}{R}p\left(x\right), \end{array}$$

with *x* = *E*/*RT* and *p*(*x*) calculated using the Senum-Yang approximation [56] of the third degree:

(6) $$\begin{array}{c} p\left(x\right)=\frac{\exp\left(-x\right)}{x}\frac{x^{2}+10x+18}{x^{3}+12x^{2}+368x+24}. \end{array}$$

The percentage error of the *p*(*x*) function for the 3^rd^ rational approximation is about 10^–5^ percent [56].

### 2.3. Kinetic Study Using the MLP Neural Network in Isothermal TG Data

The MLP neural network applied in this study was proposed initially by Sebastião et al. [48–50]. The architecture of this MLP has only one artificial neuron in the input and output layers. The intermediate layer, however, has ten artificial neurons, according to the number of kinetic models considered in the process of the thermal solid decomposition.  Table 1 presents the kinetic models studied.

The states of neurons *o*_*k*_ are defined as

(7) $$\begin{array}{c} o_{k}=f\left(\overset{m}{\underset{j=0}{\sum }}w_{kj}x_{k}\right), \end{array}$$

with *x*_*k*_ being the state of the artificial neuron in the previous layer and *w*_*kj*_ being the weights between the neurons *k* and *j*. In this particular type of network, there are also weights called bias, *w*_*i*0_, defined as unity parameters to amplify or reduce the linear correlation of the network [48, 49].

In this proposed architecture of MLP, the weights *w*_*i*1_ and the bias *w*_*i*0_ were predetermined from the fit of experimental data by each kinetic model in each studied temperature, where the weights are the rate constants and linear coefficient of the kinetic models. These weights do not change in the learning process of the network and can be represented assuming a weight matrix *w*_1_ as

(8) $$\begin{array}{c} w_{1}=\left(\begin{array}{c} w_{21}\\ w_{31}\\ w_{41}\\ \begin{array}{c} w_{51}\\ \vdots \\ w_{n1} \end{array} \end{array}\;\begin{array}{c} w_{20}\\ w_{30}\\ w_{40}\\ \begin{array}{c} w_{51}\\ \vdots \\ w_{n0} \end{array} \end{array}\right). \end{array}$$

Assuming *x* to be the experimental time data for each studied temperature,

(9) $$\begin{array}{c} x=\left(\begin{array}{c} t\\ 1 \end{array}\right). \end{array}$$

In this case, the *w*_1_*x* matrix calculates the state of the artificial neurons in the intermediate layer *W*_1_*x*:

(10) $$\begin{array}{c} w_{1}x=\left(\begin{array}{c} w_{21}t+w_{20}\\ w_{31}t+w_{30}\\ w_{41}t+w_{40}\\ \begin{array}{c} w_{51}t+w_{50}\\ \vdots \\ w_{n1}t+w_{n0} \end{array} \end{array}\right). \end{array}$$

The neurons in the intermediate layer must be activated by an activation function, *f*, to send out information to the output layer. From this, their states are defined by

(11) $$\begin{array}{c} o_{k}=f\left(w_{1}x\right). \end{array}$$

The activation functions should assume a predetermined value; generally, *f*(*x*) = 0 before calculating the state of neurons; activate the neurons assuming values near to the unity and making sure this is a increasing function *df*(*x*)/*dx* ≥ 0, to warranty the energy function optimization [49]. If we chose a linear activation function in the output layer, the network response, given by the state of the neuron in the output layer, is

(12) $$\begin{array}{c} o_{k}=w_{2}f\left(w_{1}x\right), \end{array}$$

with *W*_2_ being the interconnection weight vector of the output layer.

The MLP learning process consists of an energy function optimization, from which only the *w*_2_ vector has to be determined [49]. Considering *α*_exp_ as the experimental data, this energy function is

(13) $$\begin{array}{c} E={\left\|w_{2}f\left(w_{1}x\right)\left.-\alpha_{\exp}\right\|\right.}_{2}^{2}. \end{array}$$

To solve this equation, the *w*_2_ contribution of each kinetic model to describe the experimental data can be calculated by the well-known pseudo-inverse algorithm [49]

(14) $$\begin{array}{c} w_{2}={\left(B^{T}B\right)}^{-1}B^{T}\alpha_{\exp}, \end{array}$$

with *B* = *f*(*w*_1_*x*).

## 3. Results and Discussion

### 3.1. Thermal Decomposition of MnTE-2-PyPCl_5_

**Figure 2:**
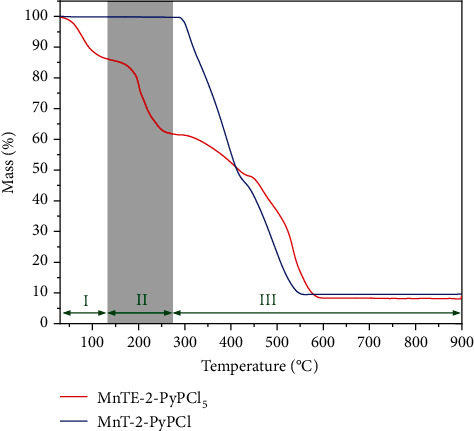
Thermogravimetric curve for MnTE-2-PyPCl_5_ (red trace, formally MnTE-2-PyPCl_5_·8H_2_O) and MnT-2-PyPCl (blue trace) under dynamic air at a heating rate of 10°C min^–1^. Thermal processes correspond to the following: I—dehydration; II—*N*-dealkylation; and III—porphyrin ring decomposition to yield Mn oxides. Corresponding data for the nonalkylated sample MnT-2-PyPCl (blue trace) is presented as a control, indicating no *N*-dealkylation process in the highlighted 134–279°C temperature range. DTG and DTA curves are included as Figures S1 and S2 in Supplementary Materials.

In our previous studies on the thermal stability of MnTE-2-PyPCl_5_ [28], we established that the thermogravimetric events of this Mn-porphyrin under dynamic air were associated with three major processes: (i) dehydration, (ii) *N*-dealkylation, and (iii) organic matter degradation to yield Mn oxide as the final residue at 900 or 950°C. The *N*-dealkylation reaction of MnTE-2-PyPCl_5_ during the thermogravimetric analyses under air at a heating rate of 10°C min^–1^ takes place between 134 and 279°C (see Figure 2). The loss of ethyl chloride (EtCl) was characterized by gas chromatography-mass spectrometry (GC-MS) of the evolved gas (EtCl) and by characterization of the residue at 279°C, which was consistent with MnT-2-PyPCl (see Figure 1) by spectroscopic and chromatographic analyses [28]. Additionally, thermogravimetric analysis of an analytical sample of MnT-2-PyPCl with no *N*-alkyl moieties (prepared independently [53]) showed no major events in the temperature range associated with EtCl loss in MnTE-2-PyPCl_5_ (see Figure 2; see also Figures S1 and S2 and Tables S1 and S2 in Supplementary Materials for complementary data by derivative thermogravimetry (DTG) and differential thermal analysis (DTA)). Whereas dehydration is a reversible process, the *N*-dealkylation of MnTE-2-PyPCl_5_ to yield MnT-2-PyPCl (see Figure 1) represents the first irreversible thermal event that compromises the MnP structural integrity, affecting permanently its catalytic efficiency, lipophilicity, bioavailability, and expected therapeutic outcome [17, 28, 38, 54]. Thus, understanding the kinetics of the MnTE-2-PyPCl_5_ thermal *N*-dealkylation process may prove useful to provide researchers and the pharmaceutic industry with information on handling/storage of MnTE-2-PyPCl_5_, shelf life, and usage. Commercially impure samples of Mn(III) 2-*N*-alkylpyridylporphyrins, containing a mixture of partially *N*-dealkylated compounds most likely derived from inadequate thermal workup procedures [38, 54], have already clouded some biological conclusions on the in vivo SOD activity of these Mn-porphyrins [54, 57].

The kinetic studies on the thermal *N*-dealkylation of MnTE-2-PyPCl_5_ were carried out by nonisothermal and isothermal protocols as presented below, observing the recommendations of the International Confederation for Thermal Analysis and Calorimetry (ICTAC) [45, 47]. The specific temperature ranges for the kinetic studies were chosen based on the interval in which only the *N*-dealkylation process of MnTE-2-PyPCl_5_ is prevalent (see Figure 2) [54]. Although thermal kinetic data are usually collected at high temperature under accelerated thermal degradation conditions, the isothermal results may be extrapolated to lower temperatures using the Arrhenius equation, to provide estimates of drug thermal stability under different target temperatures of interest to handling and storage associated with good manufacturing practices [34, 42, 58].

### 3.2. Nonisothermal Kinetic Analysis

The decomposition process associated with MnTE-2-PyPCl_5_ thermal *N*-dealkylation under nonisothermal conditions was investigated. From this methodology, the kinetic parameters of activation energy (*E*_a_) and frequency factor (*A*) were determined according to the conversion degree (*α*). The nonlinear isoconversional method proposed by Vyazovkin et al. [45] was used. This method allows an indirect analysis of multistep processes, considering the activation energy as a function of the conversion degree.

**Figure 3:**
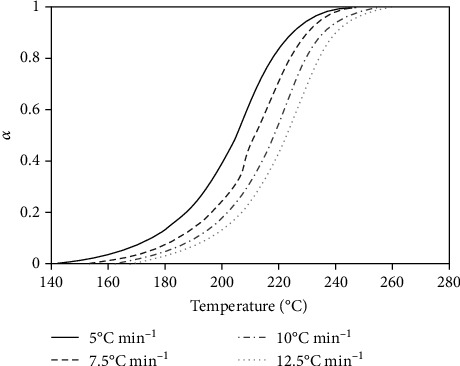
Nonisothermal curves for MnTE-2-PyPCl_5_ thermal decomposition.

**Figure 4:**
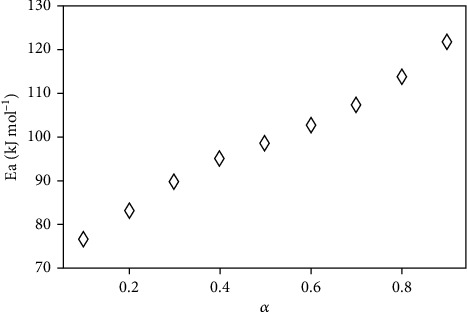
Activation energy according to the extent of conversion determined by the Vyazovkin method for the MnTE-2-PyPCl_5_ thermal *N*-dealkylation.

Figure 3 shows the nonisothermal experimental data obtained from the four heating rates chosen to follow the MnTE-2-PyPCl_5_*N*-dealkylation process: 5.0, 7.5, 10.0, and 12.5°C min^–1^. The curves are not overlapped, and observe the ICTAC recommendations for kinetic studies [45–47]. These curves were treated by the nonlinear Vyazovkin method, and Figure 4 shows the activation energy determined for the thermal decomposition process of MnTE-2-PyPCl_5_, as a function of the conversion degree. Figure 4 reveals that the activation energy increases as the conversion proceeds, starting from 75 kJ mol^–1^ at the beginning of the process, reaching 100 kJ mol^–1^ at *α* = 0.5, and finishing with values of 125 kJ mol^–1^. These high activation energies not only indicate but also are consistent with the observed high thermal stability of the compound [28]. The standard deviations for the Vyazovkin adjustments are presented in Table 2, and their small values warranty that the Vyazovkin method was used properly to treat the MnTE-2-PyPCl_5_ thermal *N*-dealkylation data. It is worth noting that the activation energy represents the energy necessary to initiate the MnTE-2-PyPCl_5_ decomposition.

### 3.3. Isothermal Kinetic Analysis by the MLP Artificial Neural Network

Isothermal decomposition of solids generally occurs at the product-reagent interface [44], being described by kinetic models well established in the literature [48–50]. These models correlate the decomposition fraction, *α*(*t*), with time at chosen appropriate temperatures. An adapted multilayer perceptron (MLP) neural network algorithm was developed by our group [48, 49] as a homemade algorithm using the MATLAB language [50, 51]. The algorithm is different from traditional MLP approaches as it does not vary the interconnection weights between the input and intermediate layers. In this MLP approach, only the weights between the intermediate and output layers are optimized, which provides the contribution of each kinetic model in the experimental data description.

**Figure 5:**
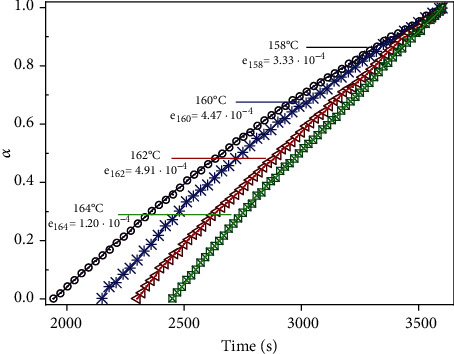
Decomposition fraction of MnTE-2-PyPCl_5_, *α*(*t*), as a function of time (*t*) at 158, 160, 162, and 164°C. Symbols are for experimental data, and the solid line is for MLP results. The residual errors (*e*) of the MLP adjustments at each temperature analyzed are also presented.

In the current study, a total of 12 kinetic models (see Table 1) were investigated simultaneously, out of which 10 models (i.e., D1, D2, D3, D4, R1, R2, R3, Am2, Am4, and Au) were found to describe in a combined manner the thermal decomposition kinetics associated with MnTE-2-PyPCl_5_ thermal *N*-dealkylation reaction. The curves of decomposition fraction, *α*(*t*), for MnTE-2-PyPCl_5_ isothermal experiments at 158, 160, 162, and 164°C are presented in Figure 5 along with the MLP adjustment considering ten kinetic models for the decomposition process. As shown in Figure 5, the residual error of the MLP adjustment in all cases is about 10^–4^, attesting the robustness of the method.

**Figure 6:**
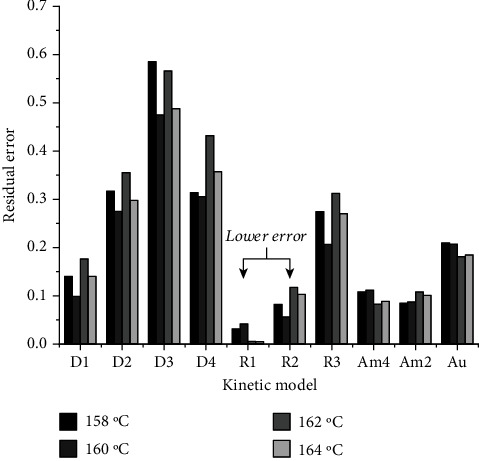
Residual errors for the adjustment of the isothermal experimental data on MnTE-2-PyPCl_5_*N*-dealkylation to each kinetic model individually.

**Figure 7:**
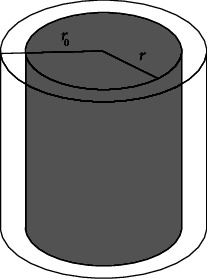
Geometrical contraction of cylinder crystals that presents the R2 kinetic mechanism.

The MLP neural network approach is exceptionally accurate compared to the adjustment of each individual kinetic model separately.  Figure 6 presents the residual error of each kinetic model considered individually to fit the experimental data. It is worth noting that fitting of experimental data with various kinetic models resulted in comparable residual errors, many of which are of the same order of magnitude (see Figure 6). This result strongly suggests that the *N*-dealkylation of MnTE-2-PyPCl_5_ via a thermal decomposition process is better understood as an overlapping contribution of many mechanisms combined [48, 49]; for this case, the R1, R2, Am2, and Am4 mechanisms presented smaller residual error to fit the experimental data. Nonetheless, the contraction models R1 and R2 explain the experimental data with more accuracy. The R2 model assumes the nucleation occurring rapidly on the surface of the crystal, which presents a cylinder shape. In this kinetic model, that accounts for a contracting area; the radius contraction follows *r* = *r*_0_ − *kt*, where *r* is the radius at time *t*, *r*_0_ is the initial radius, and *k* is the rate constant, as schematically represented in Figure 7. The generalized R*n* models, which encompass R1 and R2, are mathematical functions that describe reactions taking place in the boundary phase, with diffusion processes being extremely fast, which prevents an adequate interaction of reactants in the interface [41, 59]. These phase boundary-controlled reaction processes are, thus, controlled by the rearrangement of the reactants in the limitrophe phase. The R*n* models are known as geometrical contraction models, but R1 is similar to the ones usually applied to describe the reaction order mechanisms in a homogeneous system, following a zero-order reaction rate.

**Figure 8:**
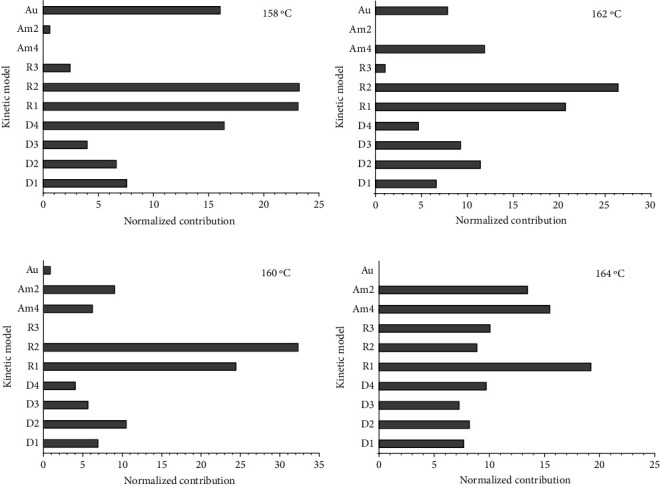
Kinetic model contributions to the description of the MnTE-2-PyPCl_5_ isothermal decomposition processes at 158, 160, 162, and 164°C (normalized values) as determined by the MLP artificial neutral network approach.

In the MLP artificial neutral network approach, ten kinetic models (i.e., D1, D2, D3, D4, R1, R2, R3, Am2, Am4, and Au, see Table 1) were considered simultaneously in the intermediate layer, with the *w*_1_ matrix (equation (8)) determined by the rate constant (*k*) and linear coefficient (*k*0) of kinetic models by the fitting experimental data of each isothermal curve. The contribution of the kinetic models to the MnTE-2-PyPCl_5_ isothermal decomposition process is presented in Figure 8, which reveals that for all studied temperatures (158, 160, 162, and 164°C), the most significant contributions were those associated with the contraction models R1 and R2. Thus, the contraction models R1 and R2 showed both smaller error and greater contribution in describing the phenomenon of MnTE-2-PyPCl_5_ thermal decomposition.

**Figure 9:**
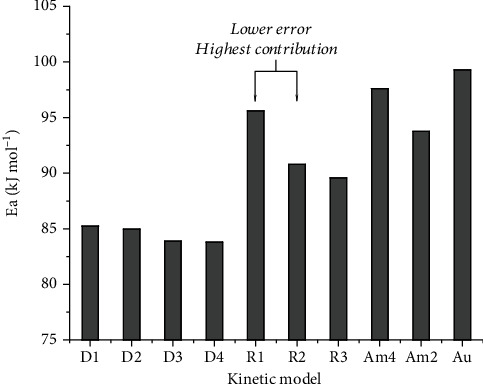
Activation energy (*E*_a_) determined by the multilayer perceptron (MLP) artificial neural network for MnTE-2-PyPCl_5_ thermal decomposition.

The rate constants determined by the experimental data adjustment into the kinetic models via the MLP neural network approach (*w*_1_ matrix, equation (8)) were used to calculate the activation energy of MnTE-2-PyPCl_5_ thermal decomposition. The MLP-derived values shown in Figure 9 are comparable to the activation energies calculated using the Vyazovkin method (see Figures 4 and 9). This agreement with the nonisothermal result indicates that the decomposition process is not affected by the experimental condition (i.e., isothermal or nonisothermal protocol) and, again, the MLP values of activation energy are consistent with the overall stability of MnTE-2-PyPCl_5_.

MnTE-2-PyPCl_5_ is often stored as a nonformulated solid or as aqueous solution formulations [9], [10, 17]. Nonformulated solid MnTE-2-PyPCl_5_ has often been stored at room temperature, presumably between 15 and 35°C [9, 10, 28]. Thus, considering the activation energy values and rate constants determined at high temperature, the kinetic parameters at 25°C were estimated, using the Arrhenius equation. These kinetic data also allowed the estimation of the *t*_90%_ shelf life value for MnTE-2-PyPCl_5_ (i.e., the time in which the initial MnP concentration decays by 10%) at 25 and 158°C (see Table 3). The *t*_90%_ value is an important parameter that is recommended for estimating the API shelf life [34, 60]. As the model R1 showed smaller error and greater contribution to the phenomenon of thermal decomposition of the material, the *t*_90%_ value found by this model is the most adequate to describe the behavior of MnTE-2-PyPCl_5_ over time. Therefore, it is expected that it would take nearly 17 years for solid MnTE-2-PyPCl_5_ to reach a 10% *N*-dealkylation at 25° C, which is consistent with the high stability empirically observed for this compound on daily routine handling and storage in the laboratory [54]. Of note, a solid sample of high-purity MnTE-2-PyPCl_5_ stored in a closed vial at room temperature in the dark for 7 years in our laboratories showed no noticeable signs of *N*-dealkylation when analyzed by thin-layer chromatography (data not shown). As shelf life values may be significantly affected by storage and drug formulations (including the nature of excipients) [34, 52, 60], the calculated *t*_90%_ value for MnTE-2-PyPCl_5_ is valid if the net solid material is kept in ideal conditions of humidity. As noted, MnTE-2-PyPCl_5_ is a hydratable molecule. The presence of humidity can trigger hydrolytic processes. High humidity generates absorption of the excess moisture in the air, which can be just as destructive as environments with low humidity. A humidity excess can compromise potency and effectiveness, leading to degradation or even toxicity in some products. It may promote the growth of microorganisms, compromising their biological integrity. The International Conference on Harmonization (ICH) in the topic Q1 A(R2) deals with the stability testing of new drugs and products, delimiting good practices of manipulation and storage concerning temperature and humidity parameters. Those variables take into account the country climate zone classification. MnTE-2-PyPCl_5_ must be stored in a hermetically sealed flask and kept at a storage temperature of 30°C ± 2°C and relative humidity of 75% ± 5% whereas Brazil belongs to climate zone IV [61, 62]. It is worth noting that these data drive further studies on MnTE-2-PyPCl_5_ formulations and stability.

## 4. Conclusions

The first study on the thermal stability kinetics of Mn(III) 2-*N*-alkylpyridylprophyrin was carried out with the prototypical MnTE-2-PyPCl_5_ compound using thermogravimetry (TG) under nonisothermal and isothermal conditions. The new multilayer perceptron (MLP) artificial neural network approach allowed the simultaneous study of ten solid-state kinetic models and showed that *N*-dealkylation associated with MnTE-2-PyPCl_5_ degradation is better explained by a combination of various mechanisms, with major contributions from the contraction models R1 and R2. The calculated activation energy values from isothermal and nonisothermal data agreed with one another and were consistent with the observed high thermal stability of MnTE-2-PyPCl_5_ in the solid state. The *t*_90%_ shelf life value at 25°C estimated from the isothermal decomposition data was approximately 17 years. These results contribute to the development of this class of redox-active therapeutics and SOD mimics and provide supporting data to protocols on purification, handling, storage, formulation, expiration date, and general use of these compounds.

Additionally, these MnTE-2-PyPCl_5_ results not only set the grounds for studying other Mn(III) porphyrin-based SOD mimics and redox-active therapeutics but also represent a first approximation to the thermal stability kinetic parameters of the chemically related Zn(II) porphyrin counterpart, ZnTE-2-PyPCl_4_, and its Zn(II) 2-*N*-alkylpyridylporphyrin analogues, which are being actively pursued as efficient and selective photosensitizers for antimicrobial photodynamic inhibition (aPDI) [63–67].

## Figures and Tables

**Table 1:** Kinetic models used in the multilayer perceptron (MLP) neural network for evaluating MnTE-2-PyPCl_5_ isothermal decomposition [43].

Kinetic model	Kinetic equation	Model type
F1	−ln(1 − *α*) = *kt* + *k*_0_	Chaotic nucleoid
Am	[−ln(1 − *α*)]^1/*m*^ = *kt* + *k*_0_, with *m* = 2, 3, 4⋯	Avrami-Erofeev
Au	$\frac{\ln\alpha }{\left(1-\alpha \right)}=kt+k_{0}$	Avrami-Erofeev
R1	*α* = *kt* + *k*_0_	Linear contraction
R2	1 − (1 − *α*)^1/2^ = *kt* + *k*_0_	Area contraction
R3	1 − (1 − *α*)^1/3^ = *kt* + *k*_0_	Volume contraction
D1	*α* ^2^ = *kt* + *k*_0_	One dimension
D2	(1 − *α*)ln(1 − *α*) + *α* = *kt* + *k*_0_	Two dimensions
D3	[1 − (1 − *α*)^1/3^]^2^ = *kt* + *k*_0_	Three dimensions
D4	$1-\frac{2}{3}\alpha -{\left(1-\alpha \right)}^{2/3}=kt+k_{0}$	Ginstling-Brounshtein

**Table 2:** Standard deviation to the Vyazovkin method according to conversion (*α*) for the MnTE-2-PyPCl_5_ thermal *N*-dealkylation.

*α* conversion	Standard deviation/10^–3^
0.10	1.1930
0.20	1.5438
0.30	2.8396
0.40	4.2336
0.50	1.5133
0.60	4.5673
0.70	3.3551
0.80	2.1008
0.90	3.8991

**Table 3:** Activation energy (*E*_a_), frequency factor as ln(*A*), and time to which the concentration decays by 10% (*t*_90%_ shelf life value), determined by the multilayer perceptron (MLP) artificial neural network for MnTE-2-PyPCl_5_ thermal decomposition at 158 and 25°C.

Kinetic model	*E*_a_ (kJ mol^–1^)	ln(*A*)	*t*_90%_ at 158°C (h)	*t*_90%_ at 25°C (years)
D1	85.2302	17.3056	59.4421	5
D2	84.9668	16.1542	69.1946	5
D3	83.9006	15.1482	141.75	9
D4	83.8007	14.5778	242.58	16
R1	95.5999	19.2077	62.05	17
R2	90.7974	17.6960	74.48	11
R3	89.5753	17.1541	91.42	12
Am4	97.6001	19.6486	69.58	25
Am2	93.7599	19.1985	37.77	8
Au	99.2897	22.0898	9.70	4

## Data Availability

The data used to support the findings of this study are available from the corresponding author upon request.

## Supplementary Materials

Supplementary Materials The thermogravimetry (TG), derivative thermogravimetry (DTG), and differential thermal analyses (DTA) were provided for both MnTE-2-PyPCl_5_ and MnT-2-PyPCl samples.
