# Supplementary material for: Thermal Stability Kinetics and Shelf Life Estimation of the Redox-Active Therapeutic and Mimic of Superoxide Dismutase Enzyme, Mn(III) meso-Tetrakis(N-ethylpyridinium-2-yl)porphyrin Chloride (MnTE-2-PyPCl5, BMX-010)
Source: Oxid Med Cell Longev. 2021 Dec 6;2021:7003861. doi: 10.1155/2021/7003861 (PMC8668311; doi:10.1155/2021/7003861)
Supplement: Supplementary Materials — The thermogravimetry (TG), derivative thermogravimetry (DTG), and differential thermal analyses (DTA) were provided for both MnTE-2-PyPCl5 and MnT-2-PyPCl samples. [file 7003861.f1.docx]

# Oxidative Medicine and Cellular Longevity

**Supplementary Materials**

# Thermal stability kinetics and shelf-life estimation of the redox-active therapeutic and mimic of superoxide dismutase enzyme, Mn(III) *meso*-tetrakis(*N*-ethylpyridinium-2-yl)porphyrin chloride (MnTE-2-PyPCl_5_, BMX-010)

Clarissa G. C. Maia,^1^ Bárbara C. R. de Araujo,^2^ Maria B. de Freitas-Marques,^2^ Israel F. da Costa,^1^ Maria Irene Yoshida,^2^ Wagner da Nova Mussel,^2^ Rita de Cássia O. Sebastião,^2^* and Júlio S. Rebouças^1^*

^1^ Departamento de Química, Centro de Ciências Exatas e da Natureza, Universidade Federal da Paraíba, João Pessoa, PB 58051-900, Brazil.

^2^ Departamento de Química, Insituto de Ciências Exatas, Universidade Federal de Minas Gerais, Belo Horizonte, MG 31207-901, Brazil.

Correspondence should be addressed to Júlio S. Rebouças; [jsreboucas@quimica.ufpb.br](mailto:jsreboucas@quimica.ufpb.br), and Rita de Cássia O. Sebastião; [ritacos@gmail.com](mailto:ritacos@gmail.com).

These Supplementary Materials provide the thermogravimetric (TG), derivative thermogravimetric (DTG), and differential thermal analyses (DTA) for both MnTE-2-PyPCl_5_ (see Figure S1 and Table S1) and MnT-2-PyPCl (see Figure S2 and Table S2) samples.

Figure S1: Thermogravimetric (TG), derivative thermogravimetric (DTG), and differential thermal analysis (DTA) curves for MnTE-2-PyPCl_5_ (formally MnTE-2-PyPCl_5_·8H_2_O) under dynamic 1 atm air at a heating rate of 10 ^o^C min^–1^.

Table S1: Thermal analysis data for MnTE-2-PyPCl_5_ (formally MnTE-2-PyPCl_5_·8H_2_O) under dynamic 1 atm air atmosphere (110 cm^3^ min^–1^) at a heating rate of 10 ^o^C min^–1^.

| **Process** | **TG/DTG** | | | | | | **DTA^a^** | **Assignment** |
| --- | --- | --- | --- | --- | --- | --- | --- | --- |
|  | Temperature,^o^C | | | | % Weight loss | | DTA peak, ^o^C |  |
|  | Range | T_onset_ | T_offset_ | DTG peak | Exp | Calcd |  |  |
| **I** | 27–134 | 57.3 | 101.1 | 77.5 | 13.9 | 12.9 | 89.7 (endo) | Loss of waters of hydration (–8 H_2_O) |
| **II** | 134–279 | 192.3 | 219.7 | 206.2 | 24.4 | 23.3 | 234.3 (endo) | Loss of EtCl  (–4 EtCl) |
| **III** |  |  |  |  |  |  |  |  |
| IIIa | 279–438 | 344.1 | 427.6 | 380.4 | 13.5 |  | 302.0 (exo) | Loss of remaining organic matter, chloride, and incorporation of oxygen to yield residual Mn oxide |
| IIIb | 438–492 | 448.7 | 477.4 | 462.1 | 9.8 |  | 439.8 (endo), 466.2 (exo) |  |
| IIIc | 492–605 | 526.5 | 560.5 | 541.7 | 29.8 |  | 493.7 (endo), 535.3 (exo) |  |
|  |  |  |  |  |  |  |  |  |
|  | 279–900 |  |  |  | 53.1 | 56.9 |  |  |
|  |  |  |  |  |  |  |  |  |
| **Residue** | 900 |  |  |  | 8.3 | 6.9 | – | Mn_3_O_4_ as final residue |

^a^ Endo stands for an endothermic event, whereas exo stands for an exothermic event.

Figure S2: Thermogravimetric (TG), derivative thermogravimetric (DTG), and differential thermal analysis (DTA) curves for MnT-2-PyPCl under dynamic 1 atm air at a heating rate of 10 ^o^C min^–1^.

Table S1: Thermal analysis data for MnT-2-PyPCl under dynamic 1 atm air atmosphere (110 cm^3^ min^‑1^) at a heating rate of 10 ^o^C min^–1^.

| **Process^a^** | **TG/DTG** | | | | | | **DTA^b^** | **Assignment** |
| --- | --- | --- | --- | --- | --- | --- | --- | --- |
|  | Temperature,^o^C | | | | % Weight loss | | DTA peak, ^o^C |  |
|  | Range | T_onset_ | T_offset_ | DTG peak | Exp | Calcd |  |  |
| **III** |  |  |  |  |  |  |  |  |
| IIIa | 285–428 | 321.0 | 335.2 | 325.7 | 53.4 |  | 326.2 (exo) | Loss of organic matter, chloride, and incorporation of oxygen to yield residual Mn oxide |
| IIIb | 428–600 | 447.0 | 455.7 | 512.0 | 36.7 |  | 502.7 (exo) |  |
|  |  |  |  |  |  |  |  |  |
|  |  |  |  |  |  |  |  |  |
|  | 285–900 |  |  |  | 90.1 | 89.2 |  |  |
|  |  |  |  |  |  |  |  |  |
| **Residue** | 900 |  |  |  | 9.5 | 10.8 | – | Mn_3_O_4_ as final residue |

^a^ Processes I (dehydration) and II (*N*-dealkylation) were absent (see Table S1).

^b^ Endo stands for an endothermic event, whereas exo stands for an exothermic event.
